# Prevalence and associated factors to alcohol use in Tunisian high school adolescents: MedSPAD 2021

**DOI:** 10.1192/j.eurpsy.2023.1835

**Published:** 2023-07-19

**Authors:** R. Mallekh, S. Rejaibi, A. Silini, M. Zid, I. Ben Slema, N. Zoghlami, S. Ben Youssef, M. Zribi, N. Ben Salah, H. Aounallah-Skhiri

**Affiliations:** 1 Department of Epidemiology, National Institute of Health; 2 Medical Faculty of Medicine, Tunis El Manar University; 3 Nutrition Surveillance and Epidemiology department, SURVEN Research Laboratory; 4 Intensive Care Unit department, Center for Urgent Medical Assistance, Tunis, Tunisia

## Abstract

**Introduction:**

Despite its well-known acute and long-term harmful effects on a person’s mental health and well-being, alcohol remains the most commonly used psychoactive substance among adolescents after tobacco products in many countries.

**Objectives:**

We aimed at studying the prevalence of alcohol use, and identify associated factors in Tunisian high school adolescents.

**Methods:**

We used national data from the 2021-Mediterranean School Survey Project on Alcohol and Other Drugs (MedSPAD). Based on a clustered two-stage stratification sampling method, a representative sample of teenagers aged 16 to 18 years, was selected. Data collection was performed using a self-administered standardized questionnaire, assessing socio-demographic characteristics and risky behaviours, and including questions about alcoholic beverages patterns of use. Binary logistic regression model was used to assess associated factors and adjusted Odds Ratios (AORs) were presented with 95% confidence intervals (CI). Cspro software was used for data entry and all statistical analysis were performed with STATA software.

**Results:**

A total of 6201 adolescents with a mean age of 16.8 years and a sex ratio female/male of 1.5 were enrolled.

Lifetime prevalence of alcoholic beverages consumption was 8.0%, 95% CI [7.0, 9,1] (n=6196). The prevalence of alcohol consumption during the previous year and month were 5.1 % and 1.7% respectively. Cocktails and beer were the most frequently consumed beverages.

Prevalence of alcohol use was significantly associated with tobacco, cannabis and e-cigarettes use (AOR 9.5, 6.0 and 1.9 respectively; p≤10^-3^), a higher frequency of nights spent away from home, school absenteeism for non-medical reasons and enrolment in the private sector.

Alcohol intoxication was reported by 2.9% of respondents during their lifetime.

Early onset was reported by 17.2% of respondents for alcohol use and 10.1% for alcohol intoxication.

**Conclusions:**

Although the prevalence of alcohol use was relatively low among Tunisian adolescents compared to European adolescents, early onset- indicating an increased risk of developing an alcohol use disorder- warrant the implementation of primary prevention interventions through mental health promotion and life skill trainings to halt these trends and avert the raising burden of morbidity and mortality attributable to alcohol use.

**Disclosure of Interest:**

None Declared

